# Identification of four novel group-specific bluetongue virus NS3 protein B-cell epitopes

**DOI:** 10.1186/s12985-015-0319-z

**Published:** 2015-06-11

**Authors:** Zhang Qin, Sun EnCheng, Xu QingYuan, Yang Tao, Wang HaiXiu, Feng YuFei, Li JunPing, Lv Shuang, Wu DongLai

**Affiliations:** The Key Laboratory of Veterinary Public Health, Ministry of Agriculture, State Key Laboratory of Veterinary Biotechnology, Harbin Veterinary Research Institute, Chinese Academy of Agricultural Sciences, Harbin, 150001 People’s Republic of China

**Keywords:** Bluetongue virus, NS3 protein, Group-specific monoclonal antibody, B-cell epitopes

## Abstract

**Background:**

The non-structural protein 3 (NS3) of bluetongue virus (BTV) is the second smaller non-structural protein produced in host cells, playing an important role in BTV trafficking and release.

**Results:**

In this study, we generated five BTV NS3-reactive monoclonal antibodies (mAbs), named 3D8, 2G9, 1B5, 4H8, and 2B12. A panel of overlapping NS3-derived peptides representing the entirety of the BTV15 NS3 protein was screened to identify linear peptide epitopes recognized by each mAb. Based on the initial screen, a series of progressively truncated peptides were produced to identify the minimal linear peptide sequence required to maintain mAb binding. We found that mAb 3D8 reacted with the motif ^36^PPRYA^40^, 2G9 reacted with the motif ^82^AEAFRDDVRLRQIK^95^, 1B5 reacted with the motif ^205^YNDAVRMSF^213^, 2B12 and 4H8 reacted with the motif ^204^SYNDAVRMSF^213^. Sequence alignments demonstrated that these linear epitopes are highly conserved among all BTV serotypes, consistent with the observation that each mAb was able to recognize cells infected with BTV1-24 serotypes tested and each identified B cell epitope was able to be recognized by BTV-infect sheep serum.

**Conclusion:**

This collection of mAbs along with defined linear epitopes may provide useful reagents for investigations of NS3 protein function and the development of BTV group-specific diagnostics.

**Electronic supplementary material:**

The online version of this article (doi:10.1186/s12985-015-0319-z) contains supplementary material, which is available to authorized users.

## Background

Bluetongue disease (BT) is a hemorrhagic disease of ruminants caused by bluetongue virus (BTV) which is spread through the bite of midges of the genus *Culicoides* [1–3]. BT is primarily found in the tropics, subtropics and temperate zones due to the limited distribution of *Culicoides* midges and presents a threat to the development of livestock farming [4–6]. Due to the severe impact of BT, the Office International Des Epizooties (OIE) lists BT as a notifiable disease.

BTV is the prototype member of the genus *Orbivirus* within the *Reoviridae* family. The BTV genome consists of 10 double-stranded RNA segments differ in lengths that encode seven structural proteins (VP1-VP7), and four non-structural protein NS1, NS2, NS3/NS3a and NS4 [7]. The BTV genome is contained in a double layer capsid. The outer virion capsid is composed of VP2 and VP5 protein, and accounts for approximately 40 % of the total protein content. The inner capsid consists of VP3 and VP7, and three secondary proteins including VP1, VP4 and VP6. Antigenic differences in the VP2 account for the different BTV serotypes, and 27 BTV serotypes are currently recognized [8, 9]. The VP2 protein elicits the generation of neutralizing antibodies *in vivo*, and is a viral hemagglutinin [10]. BTV serotypes 1, 2, 3, 4, 12, 15, and 16 are prevalent in China [11]. In addition, VP5 protein and VP2 protein can act synergistically in biological function [12]. The VP7 protein is a BTV group-specific antigen which interacts with VP3, VP2 and VP5, and plays a key role in synthesizing core particles. NS1 and NS2 are two major nonstructural proteins produced during early infection and play an essential role in virus replication [13]. The smallest non-structural protein, NS4, was recently found to play an important role in BTV virus-host interactions and may inhibit host cell antiviral responses for some BTV serotypes [14].

The shortest genome segment, BTV-S10, contains two opening reading frames (ORFs), which encodes the NS3 that has 229 amino acids and NS3a that has 216 amino acids, respectively [15]. In fact, NS3a lacks 13 amino acids at the N-terminus when compared to NS3. The BTV-S10 genome segment is highly conserved among different BTV serotypes. The non-structural proteins NS3 and NS3a produced by BTV are not components of the BTV virion itself, but are produced in host cells during BTV replication and formation [16]. NS3 first emerges from the golgi apparatus and then transferred to membrane where it primarily exists as glycosylated protein on the cytomembrane of host cells [14, 17, 18]. Some researches have highlighted differences in expression patterns of NS3/NS3a in insect cells as compared to mammalian cells [19]. NS3/NS3a is expressed at high levels in insect cells, but is expressed at much lower levels in mammalian cells where the release of BTV mainly depends on cytolysis [20–22]. Additional researches suggest that NS3 mediate BTV virion trafficking and release from infected cells *in vivo* and *in vitro* at the final stage of BTV morphogenesis with binding proteins in host cells [19, 23, 24]. However, there is a great deal that remains unknown enough about the structure and function of BTV NS3 protein. In this study, we prepared five monoclonal antibodies (mAbs) against the BTV15 NS3 protein and defined the linear epitopes recognized by each mAb. We anticipate that these reagents and results will provide a foundation for the development of BTV group-specific diagnostic technologies and facilitate studies in the structure and function of the BTV NS3 protein.

## Results

### Prokaryotic expression and purification of recombinant NS3 protein

**Fig. 1:**
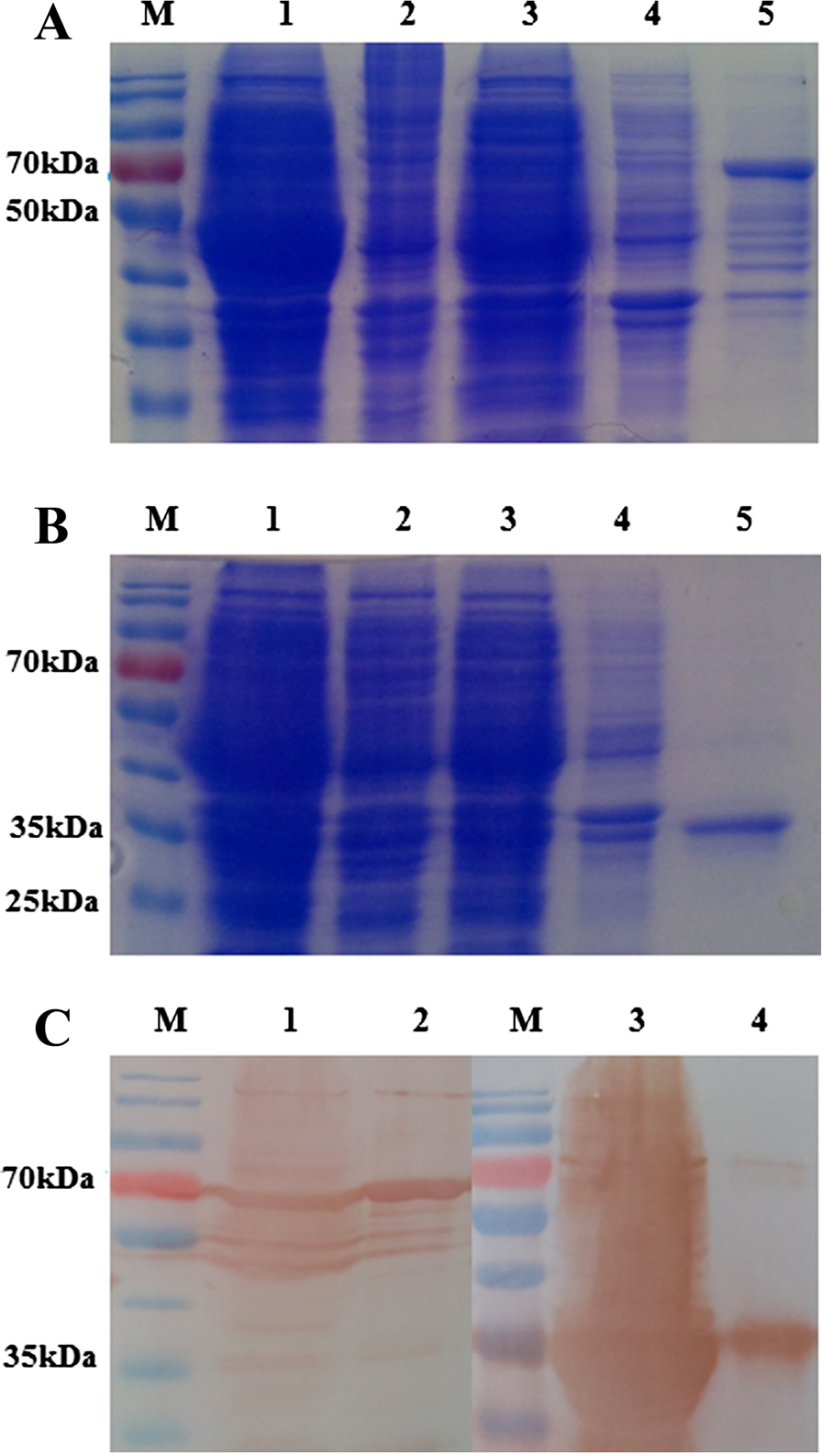
Expression and purification of recombinant BTV15-NS3 protein. **a**: SDS-PAGE analysis of recombinant MBP-NS3 protein produced in *E. coli*. M, PageRuler^TM^Prestained Protein Ladder (Fermentas, Canada); Lane 1, expression of MBP-only protein; lane 2, MBP-NS3 protein before induction; lane 3, supernatant of cell lysate of recombinant MBP-NS3 protein after ultrasonication; lane 4, cell lysate pellet of recombinant MBP-NS3 protein after ultrasonication; lane 5: purified recombinant MBP-NS3. **b**: SDS-PAGE analysis of recombinant HIS-NS3 protein in *E. coli*. M: PageRuler^TM^Prestained Protein Ladder (Fermentas, Canada); lane 1, expression of HIS-only protein; lane 2, HIS-NS3 protein before induction; lane 3, supernatant of cell lysate of recombinant HIS-NS3 protein after ultrasonication; lane 4, cell lysate pellet of recombinant HIS-NS3 protein after ultrasonication; lane 5, purified recombinant HIS-NS3. **c**: WB analysis of recombinant MBP-NS3 protein and HIS-NS3 protein. M, PageRuler^TM^Prestained Protein Ladder (Fermentas, Canada); Lane1, recombinant MBP-NS3 before purification; lane 2, purified recombinant MBP-NS3; lane 3, recombinant HIS-NS3 before purification; lane 4, purified recombinant HIS-NS3. Membranes were probed with either an HRP-conjugated anti-MBP (left panel) or anti-HIS antibody (right panel)

The recombinant NS3 protein fused with maltose-binding protein (MBP) tag (MBP-NS3) and the recombinant NS3 protein fused with a six-histidine tag (HIS-NS3) were both successfully expressed in *Escherichia coli* BL21 (DE3). MBP-NS3 was predominantly found within the soluble fraction of the induced *E. coli* after ultrasonication and was subsequently purified by amylose resin affinity chromatography (Fig. 1a). In contrast, HIS-NS3 accumulated predominantly in inclusion bodies and was therefore purified by excision of material at the appropriate molecular weight from the acrylamide gel (Fig. 1b). The two recombinant NS3 proteins were recognized by an HRP-conjugated anti-MBP mAb (Fig. 1c, left panel) and HRP-conjugated anti-histidine mAb (Fig. 1c, right panel), respectively, by Western blotting (WB).

### Preparation and identification of mAbs against BTV15 NS3 protein

We immunized BALB/c mice with purified HIS-NS3 to generate hybridoma lines secreting antibodies against the BTV15 NS3 protein. Hybridoma cell supernatants were screened by indirect enzyme-linked immunosorbent assay (ELISA) and selected hybridoma lines were subcloned by limiting dilution (data not shown). Five hybridoma lines stably producing antibodies against BTV15 NS3 protein were obtained and named 1B5, 2B12, 2G9, 3D8 and 4H8.

**Fig. 2:**
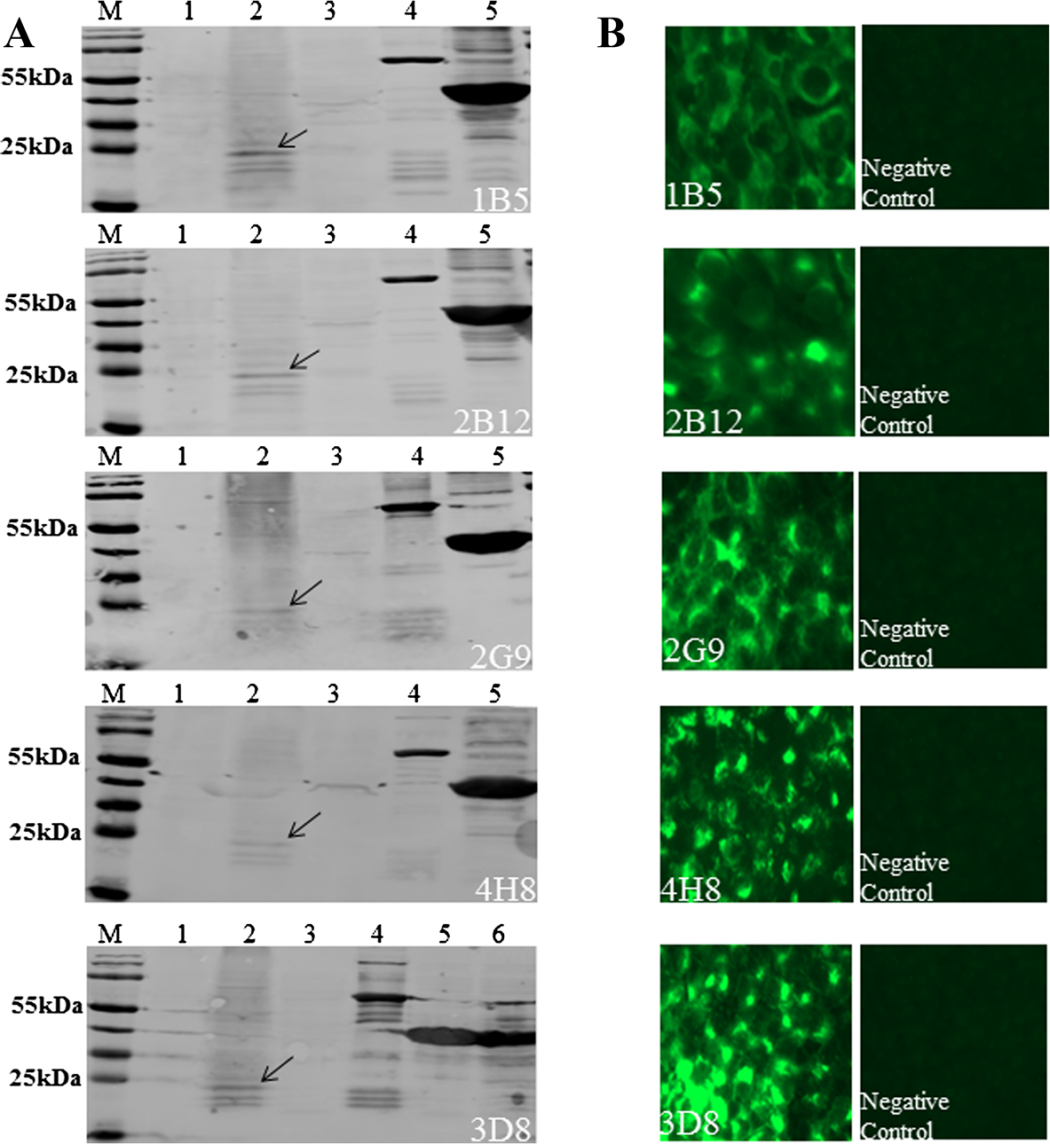
Characterization of NS3-reactive mAbs. **a**. Each mAb was tested by WB for reactivity against recombinant MBP-NS3 protein, native BTV15 NS3 protein in BTV15-infected BHK-21 cells, and its corresponding identified peptide epitope. M: PageRuler^TM^Prestained Protein Ladder; lane 1, lysate of uninfected BHK-21 cells; lane 2, lysate of BTV15-infected BHK-21; lane 3, MBP-only protein; lane 4, recombinant MBP-NS3 protein; lane 5 (and lane 6): identified linear peptide epitopes for each mAb. mAbs used are labeled at the lower right hand corner of each panel. mAb 1B5, 2B12 and 4H8 were evaluated against MBP-NS3-26; 2G9 was evaluated against MBP-NS3-11; 3D8 was evaluated against MBP-NS3-4 and MBP-NS3-5. **b**. BHK-21 cells were infected with BTV15 and used to evaluate mAb reactivity by IFA. Uninfected BHK-21 cells served as negative control for each mAb. mAbs used are labeled at the lower left hand corner of each panel

WB analysis revealed that mAbs produced by each hybridoma line reacted with the native NS3 protein derived from BTV15-infected baby hamster kidney-21 cells (BHK-21 cells) as well as the recombinant NS3 protein fused with MBP, but did not recognize uninfected BHK-21 cell lysates or the MBP fusion tag alone (Fig. 2a). In addition, each mAb was able to react with BHK-21 cells infected with BTV15 by indirect immunofluorescence assay (IFA), but did not bind appreciably to uninfected BHK-21 cells (Fig. 2b). Furthermore, each mAb also reacted with BHK-21 cells infected with all other BTV serotypes tested (BTV1–14 and BTV16–24), demonstrating that this collection of mAbs recognizes epitopes that are conserved among the BTV serotypes tested (Table 1). Whereas mAb 1B5, 2B12, 4H8, and 3D8 did not recognize BHK-21 cells infected with Chuzan virus (CV), Ibaraki virus (IBAV), or Akabane virus (AKAV), mAb 2G9 was able to additionally recognize IBAV-infected BHK-21 cells (Table 1). We could not assess the reaction of the mAbs with Epizootic hemorrhagic disease virus (EHDV) and African horse sickness virus (AHSV) as these viruses are unavailable in our laboratory at present. Each of the mAbs consisted of an IgG1 heavy chain and kappa light chain, as determined using the Mouse Monoclonal-Ab-ID kit.

**Table 1:** Analysis of reactivity of the five mAbs by IFA

mAbs	BTV1-24																								CV	IBAV	AKAV
	1	2	3	4	5	6	7	8	9	10	11	12	13	14	15	16	17	18	19	20	21	22	23	24			
1B5	+	+	+	+	+	+	+	+	+	+	+	+	+	+	+	+	+	+	+	+	+	+	+	+	−	−	−
2B12	+	+	+	+	+	+	+	+	+	+	+	+	+	+	+	+	+	+	+	+	+	+	+	+	−	−	−
2G9	+	+	+	+	+	+	+	+	+	+	+	+	+	+	+	+	+	+	+	+	+	+	+	+	−	+	−
3D8	+	+	+	+	+	+	+	+	+	+	+	+	+	+	+	+	+	+	+	+	+	+	+	+	−	−	−
4H8	+	+	+	+	+	+	+	+	+	+	+	+	+	+	+	+	+	+	+	+	+	+	+	+	−	−	−

(+) indicates a positive reaction of the mAb with BHK-21 cells infected with the indicated virus; (−) indicates no reaction of the mAb with BHK-21 cells infected with the indicated virus

*BTV* bluetongue virus, *CV* chuzan virus, *IBAV* ibaraki virus, *AKAV* akabane virus

### Identification of B-cell epitopes recognized by BTV15 NS3-reactive mAbs

**Fig. 3:**
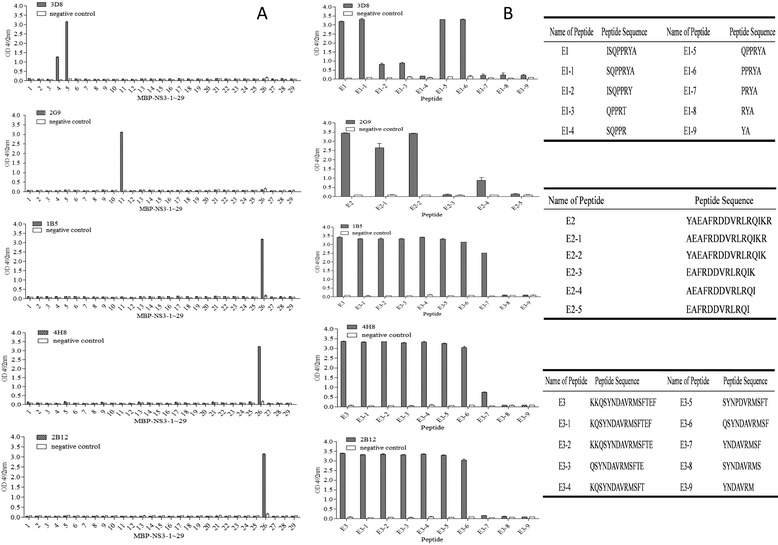
Identification of minimal linear epitopes recognized by NS3-reactive mAbs. **a**. NS3-reactive mAbs were screened by indirect ELISA against a panel of 29 overlapping peptides derived from the BTV15 NS3 amino acid sequence. The mAb used is listed in the upper left hand corner of each bar graph. A BTV15 VP2-reactive antibody was used as a negative control. The error bars display the standard deviation of three experimental repeats. **b**. mAbs were screened against a series of progressively truncated peptides based on the results of the initial screen shown in panel **a**. mAbs used are labeled on the top left corner of each bar graph. The name and the sequence of the peptides are specified in the table adjacent to each bar graph. A BTV15 VP2 protein-reactive mAb was used as a negative control antibody. The error bars display the standard deviation of three experimental repeats

We next sought to define the linear epitopes within the BTV15 NS3 protein recognized by each mAb. Peptide scanning technology was used to prepare a group of 29 overlapping MBP-fused polypeptides (MBP-NS3-1 ~ MBP-NS3-29) representing the entirety of the BTV15 NS3 protein. MBP-NS3-1 ~ MBP-NS3-29 were all successfully expressed in *E. coli* (data not shown). MBP-NS3-1 ~ MBP-NS3-29 were respectively used as coating antigen in an indirect ELISA to identify the epitopes recognized by the NS3-reactive mAbs 1B5, 2B12, 2G9, 3D8 and 4H8. Three linear epitopes within the BTV15 NS3 protein were identified (Fig. 3a). mAb 3D8 recognized both MBP-NS3-4 and MBP-NS3-5 polypeptides, suggesting that the core linear epitope was represented by the NS3-derived sequence ^33^ISQPPRYA^40^(named E1) which was the overlapping NS3 sequence present in both peptides. mAb 2G9 recognized MBP-NS3-11, which contained the NS3-derived sequence ^81^YAEAFRDDVRLRQIKR^96^ (named E2). mAbs 1B5, 2B12 and 4H8 all recognized MBP-NS3-26 which contained the NS3-derived sequence ^201^KKQSYNDAVRMSFTEF^216^ (named E3). Then, we further confirmed the results by WB (Fig. 2a). WB results showed the mAbs can react with their corresponding peptides as with the indirect ELISA results.

To define the minimal linear peptide epitopes required for mAb recognition, we designed and synthesized a series of progressively truncated polypeptides starting with the initial peptides recognized by each mAb as described above. Amino acids were progressively deleted from the N- or C-terminus of the three peptide sequences (^33^ISQPPRYA^40^, ^81^YAEAFRDDVRLRQIKR^96^ and ^201^KKQSYNDAVRMSFTEF^216^). The panel of truncated peptides was screened for mAb recognition by indirect ELISA. The minimal peptide epitope recognized by mAb 3D8 was ^36^PPRYA^40^. The minimal linear epitope recognized by 2G9 was ^82^AEAFRDDVRLRQIK^95^. mAb 1B5 recognized the minimal linear epitope ^205^YNDAVRMSF^213^, whereas 2B12 and 4H8 recognized the minimal linear epitope ^204^SYNDAVRMSF^213^ (Fig. 3b).

### Reactivity of the identified B cell epitopes with BTV-positive sheep serum

**Fig. 4:**
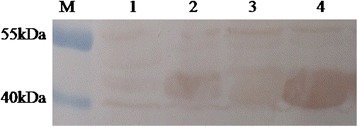
BTV-infected sheep serum recognizes the MBP-fused protein containing the amino acid sequences of identified B cell epitopes. M, PageRuler^TM^Prestained Protein Ladder (Fermentas, Canada); Lane 1, the MBP-only protein; lane 2, MBP-NS3-5 containing ISQPPRYA; lane 3, MBP-NS3-11 containing YAEAFRDDVRLRQIKR; lane 4, MBP-NS3-26 containing KKQSYNDAVRMSFTEF

To assess the reactivity of the identified B cell epitopes with BTV-positive sheep serum, the MBP-NS3-5, MBP-NS3-11 and MBP-26 respectively containing ISQPPRYA, YAEAFRDDVRLRQIKR and KKQSYNDAVRMSFTEF were used for WB analysis. The results showed BTV-positive sheep serum recognized the three identified B cell epitopes (Fig. 4 panel 2–4), but not to react with MBP-only protein (Fig. 4 panel 1). And the BTV-negative sheep serum did not react with MBP-NS3-5, MBP-NS3-11, MBP-26 or MBP-only protein (data not shown). This demonstrates that the identified B cell epitopes is targeted by sheep immune response in the context of virus infection.

### Location analysis of B cell identified epitopes in the BTV15 NS3 protein

**Fig. 5:**
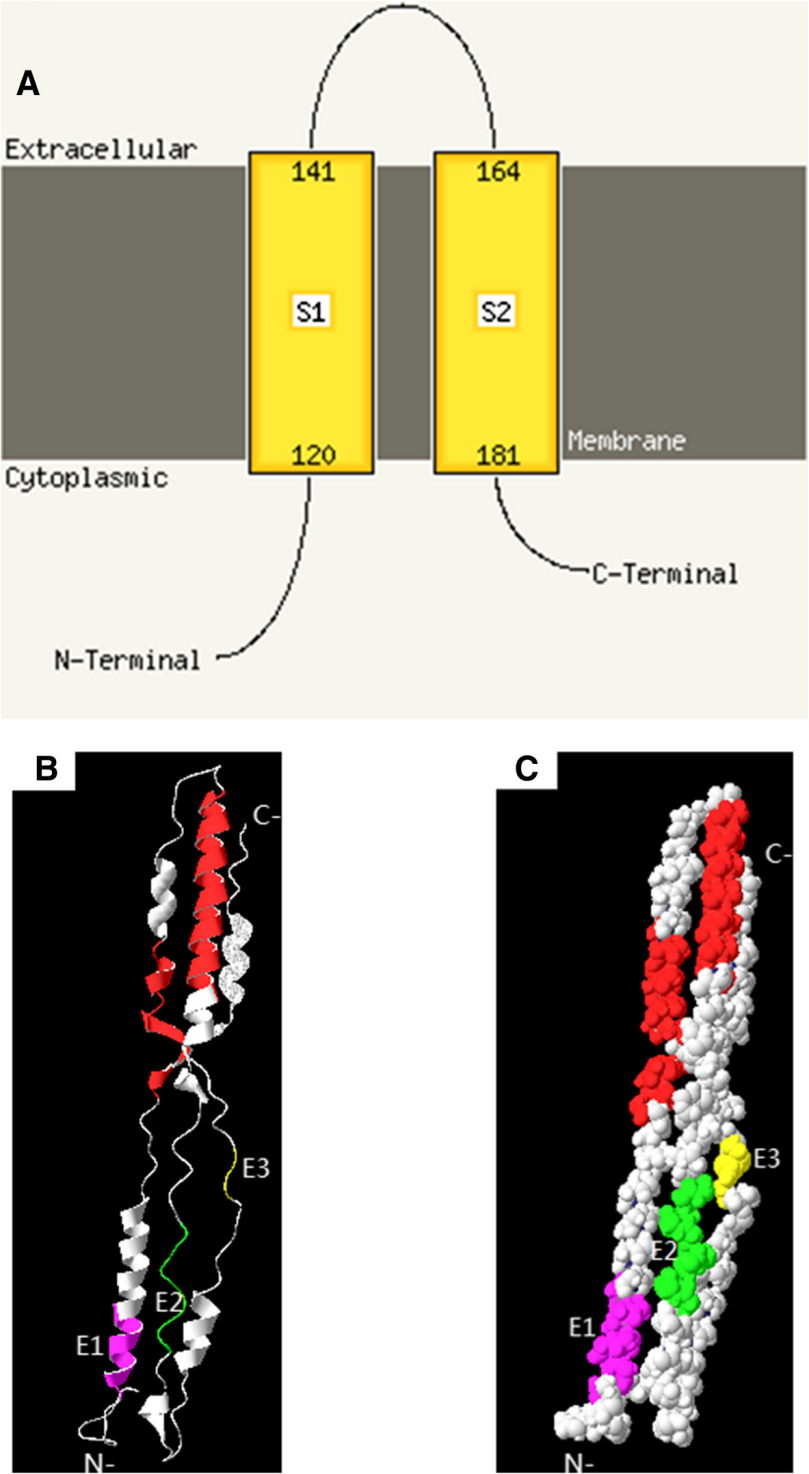
Structural analysis of the BTV15 NS3 protein and location analysis of epitopes on NS3 protein. **a**. Schematic diagram of the secondary structure of the NS3 protein using PHYRE 2 online server. The transmembrane helices regions 120-141aa and 164-181aa (S1 and S2) separated the N-terminal 1-119aa and C-terminal 182-229aa. **b** and (**c**). location analysis of the identified B cell epitopes in the BTV NS3 protein using PyMol software. 3D homology modeling was used by I-TASSER online sever. B ribbon diagram of the BTV NS3 protein; C presented the surfaces of B. the epitope E1 (^36^PPRYA^40^), E2 (^82^AEAFRDDVRLRQIK^95^) and E3 (^204^SYNDAVRMSF^213^) in B and C was labled and especially shown by yellow, green and rose red. And the transmembrane helices location(120-141aa and 164-181aa) were shown by red

Based on the data from the PHYRE 2 online sever as shown in Fig. 5a, the NS3 protein contains two transmembrane helices, located at amino acids 120–141 and 164–181 (labeled as S1 and S2 in Fig. 5a). Amino acids 142-163are exposed to the external surface of the cytoplasmic membrane, and the N-terminal amino acids 1–119 and C-terminal amino acids 182–229 exist in the cytoplasm. Therefore, the four epitopes recognized by the panel of mAbs all map to a cytoplasmic location (Fig. 5a).

Because the 3D structure of BTV NS3 protein was unavailable in Protein Data Bank (PDB), several 3D homology modeling about NS3 protein was obtained from I-TASSER online sever. Then we had chosen one of them according to high confidence that corresponding with the information of secondary structure of NS3 protein as describes above. The locations of identified epitopes were showed in the context of the deduced 3D NS3 protein model (Fig. 5b and c).

### Alignment and conversation analysis of the defined epitopes

**Fig. 6:**
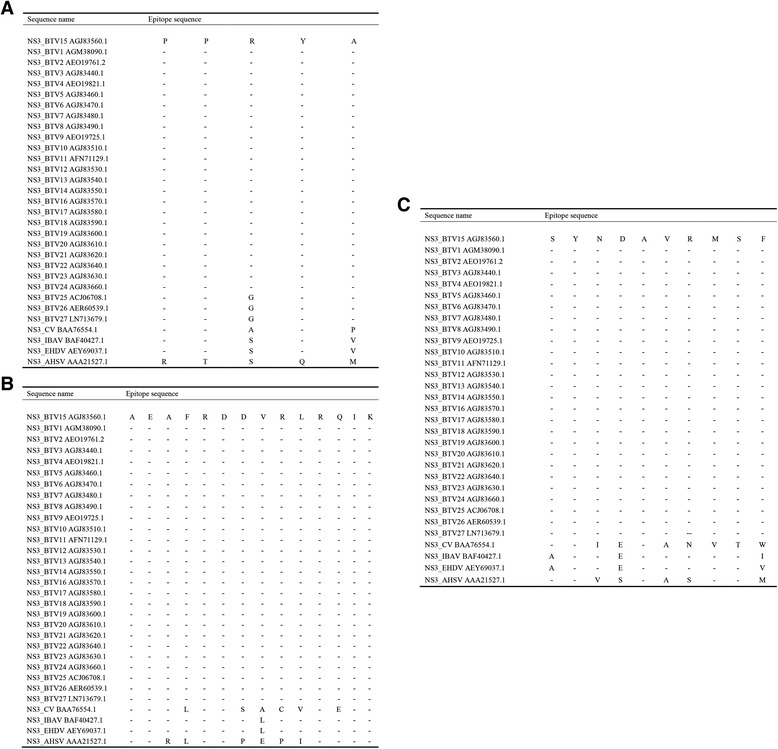
Alignment of the four identified minimal linear peptide epitopes with corresponding sequences of BTV serotypes 1–27, CV, IBAV, EHDV and AHSV. **a** Alignment of sequences corresponding to the BTV15 epitope sequence ^36^PPRYA^40^recognized by mAb 3D8; **b** Alignment of sequences corresponding to the BTV15 epitope sequence ^82^AEAFRDDVRLRQIK^95^ recognized by mAb 2G9; **c** Alignment of sequences corresponding to the BTV15 epitope sequence ^204^SYNDAVRMSF^213^ and ^205^YNPAVRMSF^213^ recognized by mAb 2B12, 1B5 and 4H8

We next analyzed the level of conservation of the four defined epitopes among different BTV serotypes and other prototype members of the genus *Orbivirus* of the family *Reoviridae.* NS3 protein sequences of viruses representing BTV serotypes 1–27, CV, IBAV, EHDV and AHSV were acquired from the UniProKB protein database. These NS3 protein sequences were aligned by using MegAlign software. The alignment results showed three defined epitopes (^82^AEAFRDDVRLRQIK^95^, ^204^SYNDAVRMSF^213^ and ^205^YNDAVRMSF^213^) were completely conserved among all 27 BTV serotypes (Fig. 6b and c). The epitope ^36^PPRYA^40^ was maintained in BTV 1–24 serotypes, whereas an R➔G substitution at position 38 was noted in BTV 25 、BTV 26 and BTV27 (Fig. 6a). In contrast, the four BTV NS3 epitopes were not as highly conserved in CV, IBAV, EHDV and AHSV. The peptide epitope ^36^PPRYA^40^ showed little similarity among CV, IBAV, EHDV and AHSV. CV, IBAV and EHDV displayed substitutions at 2 of 5 positions, and AHSV displayed substitutions at all 5 positions within this defined epitope (Fig. 6a). With respect to the BTV NS3 epitope ^82^AEAFRDDVRLRQIK^95^ (E2), CV and AHSV displayed 6 amino acid substitutions, whereas IBAV and EHDV displayed a single V➔L substitution at amino acid position 90 (Fig. 6b). The closely related linear epitopes ^204^SYNDAVRMSF^213^ (recognized by mAb 2B12 and 4H8) and ^205^YNDAVRMSF^213^ (recognized by mAb 1B5) were poorly conserved in CV and AHSV, but had higher similarity with IBAV and EHDV (Fig. 6c).

## Discussion

With the trends of global warming and the expansion of activities of *Culicoides* midges, BT disease has raised global attention with its extensively spreading. Up to date, 27 distinct BTV serotypes make diagnose and prevention of BTV more difficult and complicated. How to make group-specific diagnose of BTV quickly and efficiently is crucial. We focused on NS3 protein of BTV to develop better group-specific diagnose and research reagents. NS3 protein may represent a target for BTV group-specific diagnose because NS3 protein is highly conserved among different BTV serotypes. NS3 has been shown to possess protein-protein interaction domain that facilitate interactions with various proteins in infected cells, including cellular proteins p11, ESCRT-I protein Tsg101 and the BTV VP2 protein, involving in trafficking and release of BTV [17, 19, 25]. A study of the antibody epitopes with NS3 protein would provide the foundation for BTV group-specific diagnose and structure study of NS3 protein, even facilitate study in the contribution of NS3 on BTV trafficking and viron release by interrupting protein-protein using NS3-reactive mAbs.

In this study, we used prokaryotic recombinant protein HIS-NS3 for the immunization of BALB/c mice and MBP-NS3 for screening hybridoma supernatants for NS3-reactive antibody production to exclude potential false positive results. Five BTV-15 NS3-reactive mAbs that recognized NS3 protein produced by BTV serotypes 1–24 were generated, which demonstrate that these antibodies represent BTV group-specific mAbs.

We screened a panel of BTV15 NS3-derived peptides to map mAb reactivity to initial B cell linear epitopes. And the progressively truncated peptides were synthesized to define the minimal B cell linear epitopes recognized by these mAbs. In the initial screen, mAb 3D8 reacted with two NS3-derived peptides (MBP-NS3-4 and MBP-NS3-5) that overlapped by 8 amino acids. The binding of 3D8 to peptide MBP-NS3-5 resulted in a higher OD_492_ value when compared to 3D8 binding to MBP-NS3-4, suggesting that the overlapping sequence was sufficient for 3D8 binding, but additional residues present in MBP-NS3-5 enhanced binding. A peptide lacking the C-terminal ^40^A residue of this core peptide sequence displayed a strong reduction in 3D8 binding. When residues at the N-terminus were progressively deleted, no reduction in 3D8 binding was noted until the residue ^36^P was deleted. Therefore, we demonstrated that ^36^P and ^40^A were critical components of the linear epitope. The initial screen for 2G9 binding identified the linear epitope ^81^YAEAFRDDVRLRQIKR^96^ (E2). Deletion of ^81^Y resulted in a slight reduction in 2G9 binding, whereas deletion of ^81^YA^82^ completely abolished binding demonstrating these residues are critical components of the linear epitope. Truncation of the ^95^ K residue at the C-terminus resulted in strongly reduced 2G9 reactivity. This demonstrated that ^82^A and ^95^ K represent the critical residues for the 2G9 epitope.

The epitope ^201^KKQSYNDAVRMSFTEF^216^ (E3) was recognized by mAbs 1B5, 4H8, and 2B12. Progressive truncation of amino acid residues at the N- and C-terminus demonstrated that 1B5, 4H8, and 2B12 each retained maximal binding to the core peptide ^203^QSYNDAVRMSF^213^. Further truncation of ^203^QS^205^ strongly reduced binding of 4H8 and abolished binding of 2B12, but only slightly affected 1B5 binding. Subsequent removal of the C-terminal ^213^ F abolished 1B5, 2B12 and 4H8 binding. These results demonstrate that while 1B5, 4H8, and 2B12 recognize a common linear epitope in the BTV NS3 protein, there are slight differences in the molecular requirements for binding that are revealed through analysis of these progressively truncated peptides.

As the mAbs were derived from the mice, it is crutial if these identified B cell epitopes recognized by mAbs were actually recognized by the BTV-infected sheep serum. We used MBP-fused polypeptides containing the amino acid sequences of identified epitopes to verify the reactivity of BTV-positive sheep serum with the MBP-fused polypeptides. The result showed BTV-positive sheep serum was able to recognized all of the identified B cell epitopes, demonstrating the practicability for the BTV group-specific diagnostic system.

Based on previous research [23, 26, 27], we also used the bioinformatic software PHYRE 2 online sever to analyze NS3 secondary structure and mapped the locations of identified epitopes on 3D homology model of NS3 protein in this study. we deduced that the NS3 protein exists as a transmembrane protein in host cells and has two helices that span double-layer membranes. The N- and C-terminal ends of the NS3 protein exist in the cytoplasm. In addition, some residues are exposed to the external surface of the cytoplasmic membrane. In this accord, the B-cell epitopes recognized by these five mAbs are all predicted to be located in the cytoplasm of host cells. Because there is no NS3 crystal structure available now, the I-TASSER online sever was used to predict the 3D structure based on different templates of high confidence by sending the primary sequence of NS3. The analysis of NS3 protein structure could potentially facilitate the function study of the NS3 protein.

Alignment of NS3 amino acid sequences of different BTV subtypes and select prototype members of the genus *Orbivirus* of the family *Reoviridae*(CV, IBAV, EHDV and AHSV), showed that the defined antibody epitopes are highly conserved among BTV serotypes, but less conserved among other *orbiviruses*. This analysis was consistent with the IFA results which demonstrated that the mAbs reacted with BHK-21 cells infected with all BTV serotypes tested (1–24), but not with CV-, IBAV-, or AKAV-infected cells. One exception was mAb 2G9, which was able to react with IBAV-infected BHK-21 cells in addition to BTV-infected BHK-21 cells. We infer that this is because the BTV epitope ^82^AEAFRDDVRLRQIK^95^recognized by 2G9 differs by only a valine-to-leucine substitution in the NS3 protein of IBAV.

## Conclusions

In summary, we generated five BTV NS3 reactive mAbs and defined four novel linear B-cell epitopes on the NS3 protein of BTV15. The generation of this panel of NS3-specific mAbs with defined linear peptide epitopes may serve as a foundation for studies aiming to elucidate the role of NS3 protein-protein interactions on virus replication and may be useful reagents for the development of BTV group-specific diagnostic system.

## Materials and methods

### Cell lines and viruses

BHK-21 cells and the SP2/0 myeloma cell line were cultured in Dulbecco’s Modified Eagle’s Medium (DMEM, Invitrogen) in a humidified incubator with 5 % CO_2_ at 37 °C. Media for BHK-21 cells and SP2/0 myeloma cells were respectively supplemented with 10 and 20 % heated-inactivated fetal bovine serum (GIBCO, Invitrogen) along with antibiotics (0.1 mg/ml of streptomycin and 100 IU/ml of penicillin). BTV serotypes 1–24 and CV, IBAV and AKAV were maintained at the Harbin Veterinary Research Institute and were propagated in BHK-21 cells using standard techniques.

### Prokaryotic expression of recombinant NS3 protein

The full length of the BTV15 S10 gene segment encoding the NS3 protein (GenBank accession number AGJ83560.1) was amplified from the pEASY-NS3 plasmid (available in our laboratory) using the following primer pair:

BTV15 NS3-*Eco*RI-F-1 ~ 19:5′-CC*GAATTC*ATGCTATCCGGGCTGATCC-3′and

BTV15 NS3-*Hin*dIII-R-671 ~ 690:5′-GC*AAGCTT*TCAGGTTAATGGCATTTCGA-3′

Primers included *Eco*RI and *Hin*dIII restriction sites (shown in italics) at the 5′end to facilitate directional cloning into the pMAL^TM^-c4X expression vector (New England Biolab Inc., USA) and the pET-30a (+) expression vector (Novagen, USA). Plasmid construction was verified by restriction enzyme digestion, PCR, and DNA sequencing. Cloning into the pMAL^TM^-c4X expression vector placed the NS3 coding sequence downstream from the malE gene, which encodes MBP and facilitates expression of an MBP-BTV15 NS3 fusion protein. Similarly, cloning into the pET-30a (+) expression vector facilitates expression of BTV15 NS3 fused with a N-terminal six-histidine tag. The two recombinant plasmids, named pC4X-NS3-BTV15 and pET-NS3-BTV15, were transformed to *E. coli* to produce MBP-BTV15 NS3 protein and HIS-BTV15 NS3 protein, respectively. Recombinant NS3 protein was induced by adding isopropyl-D-thiogalactopyranoside (IPTG). For MBP-BTV15 NS3 protein expression, IPTG was added to a final concentration of 1.0 mM and cells were incubated overnight at 16 °C. For HIS-BTV15 NS3 expression, IPTG was added to a final concentration of 0.75 mM and cells were incubated for 6 h at 37 °C. Production of recombinant NS3 protein was evaluated by sodium dodecylsulfate-polyacrylamide gel electrophoresis (SDS-PAGE). The HIS-NS3 protein was purified by excision of the protein-containing band from the acrylamide gel. MBP-NS3 protein was purified by affinity chromatography using an amylose resin according to the instructions of the pMAL^TM^Protein Fusion and Purification System, Version 5.01 (NEW England Biolabs Inc., USA). Production of the two purified recombinant BTV15 NS3 proteins was confirmed by WB as follows. Recombinant proteins were boiled 5–10 min, separated by SDS-PAGE, and transferred to nitrocellulose membranes. The membranes were blocked with 5 % skim milk overnight at 4 °C to reduce non-specific binding. To detect MBP-BTV15 NS3 and HIS-BTV15 NS3, a 1:4000 dilution of an HRP-conjugated anti-MBP mAb (New England Biolab Inc., USA) or an HRP-conjugated anti-HIS mAb (New England Biolab Inc., USA) was added to each respective membrane. Membranes were incubated at room temperature for 1 h and visualized following the addition of 3,3′-diaminobenzidine tetrahydrochloride (DAB; ZSGB-BIO, China) [28]. Reactions were stopped by washing with deionized water before being photographed.

### Preparation and identification of mAbs against BTV15 NS3 protein

Firstly, we crushed down the gel contained the purified HIS-BTV15-NS3 protein and acutely stirred till it presented mushy state. Then, six-week-old female BALB/c mice were immunized subcutaneously with 100 μg HIS-BTV15 NS3 protein as above. After 14 days, mice received a subcutaneous booster immunization consisting of 200 μg HIS-BTV15 NS3 protein. The mice received a final booster immunization 3 days prior to harvesting splenocytes for hybridoma generation. Splenocytes were fused with SP2/0 myeloma cells at a ratio of 4:1 using polyethylene glycol (PEG4000, Sigma-Aldrich). The cells were seeded into 96-well plates in HAT medium (DMEM containing 20 % FBS, 100 μg/ml streptomycin,100 IU/ml penicillin,100 mM hypoxanthine, 16 mM thymidine, and 400 mM aminopterin) for 6 days at which time the media was replaced with HT medium (HAT medium as above lacking aminopterin) [29]. Hybridoma lines producing NS3-reactive antibodies were sub-cloned at least 3 times by limiting dilution. Hybridoma supernatants were screened by indirect ELISA, WB and IFA as previously described [29, 30].

Briefly, for ELISA, 96 well plates were sensitized at 4 °C overnight with purified MBP-NS3 as coating antigen at 100 ng/ml. After blocked with 5 % skim milk at 37 °C for 2 h, the plates were incubated with hybridoma supernatants at 37 °C for 1 h, then with an HRP-conjugated goat anti-mouse secondary antibodies at a 1:4000 dilution at 37 °C for 1 h. Finally, the substrate solution containing ophenylenediamine (OPD) was used as color development to evaluate the antigen-antibody reaction result.

In WB, the hybridoma supernatants were used as primary antibodies and an HRP-conjugated goat antibody was used as secondary antibody.

For IFA, the 96 well microtiter plates were respectively added with BTV1-24, CV, IBAV and AKAV. About 24–48 h later, BHK-21 cells were fixed with 90 % (V/V) ethylalcohol in precooled distilled water at 4 °C for 30 min. The cells were washed with phosphate buffered saline (PBS, pH7.4) with 0.1 % Tween-20 (Sigma, USA) and were added in hybridoma supernatants for incubating at 37 °C for 1 h. The plates were washed again and a FITC-conjugated goat anti-mouse IgG (Sigma, USA) was added as secondary antibody. Finally, the plates were viewed by a fluorescence microscope after three times of additional washes.

NS3-reactive mAbs subtypes were determined using the Mouse Monoclonal-Ab-ID kit (SBA Clonotyping System-HRP, SouthernBiotech) as the manufacturer’s instructions.

### Expression of BTV15 NS3-derived polypeptides

In order to map the epitopes of NS3-reactive mAbs, we designed and produced a panel of 29 overlapping polypeptides named MBP-NS3-1 ~ MBP-NS3-29 covering the entirety of the BTV15 NS3 protein. Each polypeptide of MBP-NS3-1 ~ MBP-NS3-28 in the series was 16 amino acids in length and overlapped with adjacent peptides by 8 amino acids, for example, the first peptide corresponded to NS3 amino acids 1–16 and the second peptide corresponded to NS3 amino acids 9–24. But the last one of MBP-NS3-29 was 5 amino acids that overlapped with MBP-NS3-28 in order to ensure that potential linear epitopes were represented in the NS3 peptide series. To generate the peptide series, we synthesized 29 pairs of complementary oligonucleotides encoding each peptide sequence (Additional file 1: Table S1). Oligonucleotides also incorporated a TGA termination codon and *Eco*RI and *Hin*dIII restriction sites to facilitate cloning into the pMAL^TM^-c4X expression plasmid, which places the peptide sequence in-frame with a MBP coding sequence. Each peptide-encoding plasmid was transformed into *E. coli* BL21 (DE3) (Novagen, USA) for expression. Production of each MBP-fused recombinant polypeptide was confirmed by SDS-PAGE.

### Epitope identification

To identify linear epitopes recognized by each mAb, the mAbs produced as described above were initially screened against the MBP-fused recombinant polypeptide series by indirect ELISA and confirmed via WB. MBP-fused NS3 peptides and an MBP-only protein control were coated as target antigen in 96 well plates. mAbs were added as the primary antibody, and an HRP-conjugated goat anti-mouse IgG (ZSGB-BIO, China) was used as the secondary antibody. Absorbance readings were taken following addition of a substrate solution containing OPD. Based on the results of this initial screen used to identify a linear region of NS3 recognized by each mAb, we designed and synthesized a series of progressively truncated polypeptides to perform a more refined mapping of the minimal linear peptide epitope recognized by each mAb using the approaches described above.

### Detection of the reactivity of the epitopes with BTV-positive sheep serum

To investigate whether the epitopes could be detected by BTV-positive sheep serum, the MBP-fused polypeptides containing PPRYA, AEAFRDDVRLRQIK, SYNDAVRMSF (MBP-NS3-5, MBP-NS3-11 and MBP-NS3-26 were used here) were used to react with BTV-positive sheep serum by WB. The WB procedure was the same as described above. BTV-positive sheep serum available in our laboratory was performed as the primary antibody and HRP-conjugated rabbit anti-sheep was the secondary antibody (LICOR Biosciences). A BTV-negative sheep serum available in our laboratory was also been tested as a control with the same processes.

### Location analysis of identified epitopes in the BTV15 NS3 protein

We used the bioinformatic software PHYRE 2 online sever to analyze NS3 secondary structure [31]. In order to locate and explain the general spatial relationship of epitopes on NS3 protein according to the homology modeling from PDB, a 3D model of NS3 protein was generated by using I-TASSER online sever [32, 33]. And the PyMol software was used based on the results of I-TASSER online sever to analyze the epitopes locations onto NS3 3D model.

### Homology analysis of defined epitopes

We assessed the level of conservation of each identified BTV15 NS3 linear epitope among representative viruses of BTV serotypes 1–27 as well as prototype members of the genus *Orbivirus* in the family *Reoviridae*, including CV, IBAV, EHDV and AHSV. For each virus, we identified the amino acid sequence corresponding to the identified BTV15 NS3 epitope using UniProKB protein database (http://www.uniprot.org/) and aligned the sequences for comparison using the analytical software MegAlign (Lasergene, DNASTAR Inc., Madison, WI, USA).

### Statistical analysis

The software GraphPad Prism 5 was used for statistical evaluation.

## Additional file

Additional file 1: Table S1. Designation of 29 pairs of complementary oligonucleotides.
