# Supplementary material for: Identification of four novel group-specific bluetongue virus NS3 protein B-cell epitopes
Source: Virol J. 2015 Jun 11;12:86. doi: 10.1186/s12985-015-0319-z (PMC4514961; doi:10.1186/s12985-015-0319-z)
Supplement: Additional file 1: Table S1. — Designation of 29 pairs of complementary oligonucleotides. [file 12985_2015_319_MOESM1_ESM.docx]

Additional file 1: Table S1. Designation of 29 pairs of complementary oligonucleotides

| Designations  of oligonucleotides | The sequences of oligonucleotides  (5’→3’) | Coding peptides |
| --- | --- | --- |
| B15-NS3-1-F | AATTCATGCTATCCGGGCTGATCCAAAGGTTCGAAGAAGAAAAGATGAAACACTGAA | MLSGLIQRFEEEKMKH |
| B15-NS3-1-R | AGCTTTCAGTGTTTCATCTTTTCTTCTTCGAACCTTTGGATCAGCCCGGATAGCATG |  |
| B15-NS3-2-F | AATTCTTCGAAGAAGAAAAGATGAAACACAATCAAAATAGAGTTGAAGAGCTGTGAA | FEEEKMKHNQNRVEEL |
| B15-NS3-2-R | AGCTTTCACAGCTCTTCAACTCTATTTTGATTGTGTTTCATCTTTTCTTCTTCGAAG |  |
| B15-NS3-3-F | AATTCAATCAAAATAGAGTTGAAGAGCTGAGTTTAGTGCGTGTGGATGATACTTGAA | NQNRVEELSLVRVDDT |
| B15-NS3-3-R | AGCTTTCAAGTATCATCCACACGCACTAAACTCAGCTCTTCAACTCTATTTTGATTG |  |
| B15-NS3-4-F | AATTCAGTTTAGTGCGTGTGGATGATACTATCTCTCAACCACCAAGGTATGCTTGAA | SLVRVDDTISQPPRYA |
| B15-NS3-4-R | AGCTTTCAAGCATACCTTGGTGGTTGAGAGATAGTATCATCCACACGCACTAAACTG |  |
| B15-NS3-5-F | AATTCATCTCTCAACCACCAAGGTATGCTCCGAGTGCACCGATGCCATCGTCTTGAA | ISQPPRYAPSAPMPSS |
| B15-NS3-5-R | AGCTTTCAAGACGATGGCATCGGTGCACTCGGAGCATACCTTGGTGGTTGAGAGATG |  |
| B15-NS3-6-F | AATTCCCGAGTGCACCGATGCCATCGTCTATGCCAACAGTTGCCCTTGAAATATGAA | PSAPMPSSMPTVALEI |
| B15-NS3-6-R | AGCTTTCATATTTCAAGGGCAACTGTTGGCATAGACGATGGCATCGGTGCACTCGGG |  |
| B15-NS3-7-F | AATTCATGCCAACAGTTGCCCTTGAAATATTGGACAAAGCGATGTCAAACACATGAA | MPTVALEILDKAMSNT |
| B15-NS3-7-R | AGCTTTCATGTGTTTGACATCGCTTTGTCCAATATTTCAAGGGCAACTGTTGGCATG |  |
| B15-NS3-8-F | AATTCTTGGACAAAGCGATGTCAAACACAACTGGTGCAACGCAAACGCAAAAATGAA | LDKAMSNTTGATQTQK |
| B15-NS3-8-R | AGCTTTCATTTTTGCGTTTGCGTTGCACCAGTTGTGTTTGACATCGCTTTGTCCAAG |  |
| B15-NS3-9-F | AATTCACTGGTGCAACGCAAACGCAAAAAGCGGAGAAAGCTGCATTCGCATCGTGAA | TGATQTQKAEKAAFAS |
| B15-NS3-9-R | AGCTTTCACGATGCGAATGCAGCTTTCTCCGCTTTTTGCGTTTGCGTTGCACCAGTG |  |
| B15-NS3-10-F | AATTCGCGGAGAAAGCTGCATTCGCATCGTACGCGGAAGCGTTTCGTGATGATTGAA | AEKAAFASYAEAFRDD |
| B15-NS3-10-R | AGCTTTCAATCATCACGAAACGCTTCCGCGTACGATGCGAATGCAGCTTTCTCCGCG |  |
| B15-NS3-11-F | AATTCTACGCGGAAGCGTTTCGTGATGATGTAAGATTGAGACAGATTAAGCGCTGAA | YAEAFRDDVRLRQIKR |
| B15-NS3-11-R | AGCTTTCAGCGCTTAATCTGTCTCAATCTTACATCATCACGAAACGCTTCCGCGTAG |  |
| B15-NS3-12-F | AATTCGTAAGATTGAGACAGATTAAGCGCCATGTGAATGAGCAGATTTTACCTTGAA | VRLRQIKRHVNEQILP |
| B15-NS3-12-R | AGCTTTCAAGGTAAAATCTGCTCATTCACATGGCGCTTAATCTGTCTCAATCTTACG |  |
| B15-NS3-13-F | AATTCCATGTGAATGAGCAGATTTTACCTAAATTAAAAGGTGATCTAAGTGGATGAA | HVNEQILPKLKGDLSG |
| B15-NS3-13-R | AGCTTTCATCCACTTAGATCACCTTTTAATTTAGGTAAAATCTGCTCATTCACATGG |  |
| B15-NS3-14-F | AATTCAAATTAAAAGGTGATCTAAGTGGATTGAAGAAGAAGCGAGCGATCATATGAA | KLKGDLSGLKKKRAII |
| B15-NS3-14-R | AGCTTTCATATGATCGCTCGCTTCTTCTTCAATCCACTTAGATCACCTTTTAATTTG |  |
| B15-NS3-15-F | AATTCTTGAAGAAGAAGCGAGCGATCATACATACTACTCTACTGGTAGCTGCCTGAA | LKKKRAIIHTTLLVAA |
| B15-NS3-15-R | AGCTTTCAGGCAGCTACCAGTAGAGTAGTATGTATGATCGCTCGCTTCTTCTTCAAG |  |
| B15-NS3-16-F | AATTCCATACTACTCTACTGGTAGCTGCCGTGGTTGCGCTATTAACATCAGTTTGAA | HTTLLVAAVVALLTSV |
| B15-NS3-16-R | AGCTTTCAAACTGATGTTAATAGCGCAACCACGGCAGCTACCAGTAGAGTAGTATGG |  |
| B15-NS3-17-F | AATTCGTGGTTGCGCTATTAACATCAGTTTGCACCCTTTCAAGCGATATGAGTTGAA | VVALLTSVCTLSSDMS |
| B15-NS3-17-R | AGCTTTCAACTCATATCGCTTGAAAGGGTGCAAACTGATGTTAATAGCGCAACCACG |  |
| B15-NS3-18-F | AATTCTGCACCCTTTCAAGCGATATGAGTGTGGCCTTCAAGATAAATGGGACTTGAA | CTLSSDMSVAFKINGT |
| B15-NS3-18-R | AGCTTTCAAGTCCCATTTATCTTGAAGGCCACACTCATATCGCTTGAAAGGGTGCAG |  |
| B15-NS3-19-F | AATTCGTGGCCTTCAAGATAAATGGGACTAAGACAGAAGTGCCTTCATGGTTTTGAA | VAFKINGTKTEVPSWF |
| B15-NS3-19-R | AGCTTTCAAAACCATGAAGGCACTTCTGTCTTAGTCCCATTTATCTTGAAGGCCACG |  |
| B15-NS3-20-F | AATTCAAGACAGAAGTGCCTTCATGGTTTAAAAGCCTTAATCCGATGCTTGGCTGAA | KTEVPSWFKSLNPMLG |
| B15-NS3-20-R | AGCTTTCAGCCAAGCATCGGATTAAGGCTTTTAAACCATGAAGGCACTTCTGTCTTG |  |
| B15-NS3-21-F | AATTCAAAAGCCTTAATCCGATGCTTGGCGTTGTCAATTTGGGAGCAACTTTTTGAA | KSLNPMLGVVNLGATF |
| B15-NS3-21-R | AGCTTTCAAAAAGTTGCTCCCAAATTGACAACGCCAAGCATCGGATTAAGGCTTTTG |  |
| B15-NS3-22-F | AATTCGTTGTCAATTTGGGAGCAACTTTTTTGATGATGGTTTGCGCAAAGAGTTGAA | VVNLGATFLMMVCAKS |
| B15-NS3-22-R | AGCTTTCAACTCTTTGCGCAAACCATCATCAAAAAAGTTGCTCCCAAATTGACAACG |  |
| B15-NS3-23-F | AATTCTTGATGATGGTTTGCGCAAAGAGTGAAAGAGCTTTGAATCAGCAGATATGAA | LMMVCAKSERALNQQI |
| B15-NS3-23-R | AGCTTTCATATCTGCTGATTCAAAGCTCTTTCACTCTTTGCGCAAACCATCATCAAG |  |
| B15-NS3-24-F | AATTCGAAAGAGCTTTGAATCAGCAGATAGATATGATAAAGAAAGAAGTGATGTGAA | ERALNQQIDMIKKEVM |
| B15-NS3-24-R | AGCTTTCACATCACTTCTTTCTTTATCATATCTATCTGCTGATTCAAAGCTCTTTCG |  |
| B15-NS3-25-F | AATTCGATATGATAAAGAAAGAAGTGATGAAAAAACAATCTTATAACGATGCGTGAA | DMIKKEVMKKQSYNDA |
| B15-NS3-25-R | AGCTTTCACGCATCGTTATAAGATTGTTTTTTCATCACTTCTTTCTTTATCATATCG |  |
| B15-NS3-26-F | AATTCAAAAAACAATCTTATAACGATGCGGTGAGGATGAGTTTTACAGAGTTCTGAA | KKQSYNDAVRMSFTEF |
| B15-NS3-26-R | AGCTTTCAGAACTCTGTAAAACTCATCCTCACCGCATCGTTATAAGATTGTTTTTTG |  |
| B15-NS3-27-F | AATTCGTGAGGATGAGTTTTACAGAGTTCTCGTCAGTCCCGCTGGATGGTTTCTGAA | VRMSFTEFSSVPLDGF |
| B15-NS3-27-R | AGCTTTCAGAAACCATCCAGCGGGACTGACGAGAACTCTGTAAAACTCATCCTCACG |  |
| B15-NS3-28-F | AATTCTCGTCAGTCCCGCTGGATGGTTTCGAAATGCCATTAACCTGAA | SSVPLDGFEMPLT |
| B15-NS3-28-R | AGCTTTCAGGTTAATGGCATTTCGAAACCATCCAGCGGGACTGACGAG |  |
| B15-NS3-29-F | AATTCGAAATGCCATTAACCTGAA | EMPLT |
| B15-NS3-29-R | AGCTTTCAGGTTAATGGCATTTCG |  |
